# Nicotine Contents in Some Commonly Used Toothpastes and Toothpowders: A Present Scenario

**DOI:** 10.1155/2012/237506

**Published:** 2012-01-12

**Authors:** S. S. Agrawal, R. S. Ray

**Affiliations:** Department of Pharmacology, Delhi Institute of Pharmaceutical sciences and Research (DIPSAR), University of Delhi, Pushp Vihar, Sector 3, M.B. Road, New Delhi 110017, India

## Abstract

The use of tobacco products as dentifrices is still prevalent in various parts of India. Tobacco use in dentifrices is a terrible scourge which motivates continued use despite its harmful effects. Indian legislation prohibits the use of nicotine in dentifrices. Nicotine is primarily injurious to people because it is responsible for tobacco addiction and is dependence forming. The present study was motivated by an interest in examining the presence of nicotine in these dentifrices. Our earlier report indicates the presence of nicotine in toothpowders. To further curb the menace of tobacco, our team again analysed the toothpowder brands of previous years and in toothpastes as well. Eight brands of commonly used toothpastes and toothpowders were evaluated by gas chromatography-mass spectroscopy. On the whole, there are a few successes but much remains to be done. Our findings indicated the presence of nicotine in two brands of *dant manjans* and four brands of toothpastes. Further our finding underscores the need for stringent regulations by the regulatory authorities for preventing the addition of nicotine in these dentifrices. Hence government policy needs to be targeted towards an effective control of tobacco in these dentifrices and should be properly addressed.

## 1. Introduction

Nicotine [1-methyl-2-(3-pyridyl-pyrrolidine), C_10_H_14_N_2_] is the principal component of tobacco [1]. The predominant effects of nicotine include rise in blood pressure, heart rate, respiratory rate; increase in the level of catecholamines in blood; increase in level of free fatty acids, mobilization of blood sugar, and it was also found to disturb the antioxidant defense mechanisms [2–6]. There is strong evidence that smokeless tobacco use leads to oral mucosal lesions [7, 8] including oral pre-cancerous lesions, gingival recession [9], cardiovascular risk factors and disease, diabetes, reproductive health effects, and overall mortality. Nicotine rapidly crosses the placenta and has toxic effects on the foetus [10, 11]. Regular tobacco use reduces life expectancy by approximately 7 years [12].

India's share of the global burden of tobacco-induced disease and death is substantial [13]. India has one of the highest rates of oral cancers in the world; 65% of all cancers in men and 33% of all cancers in women are tobacco related. Annual incidence of oral cancer is said to be 10 per 1,00,000 per males [14].

In India, there is a widespread misconception that tobacco is good for the teeth [15]. Tobacco products are popular as a dentifrice in different parts of India and children also use such dentifrices [16]. Among female smokeless tobacco users, the dominating form is tobacco toothpowder (41.3%). Among men it was khaini (57.1%) followed by tobacco toothpowder (8.8%) [16, 17]. Many companies take advantage of this misconception and exploiting the addictive nature of nicotine by packaging and positioning their products as dental care products without explicitly stating so. 

There was an amendment in Drugs & Cosmetics Act, 1940 vide notification published in the Gazette of India vide G.S.R. 443(E) and 444(E) dated 30.04.1992, mentioning that manufacture and sale of all cosmetics and all Ayurvedic drugs licensed as toothpowders/toothpastes containing tobacco have been prohibited [6, 18]. However, recent studies reported the presence of nicotine in some toothpowders [6, 18].

The present studies were motivated by an interest in examining the presence of nicotine in these dentifrices to evaluate the presence of nicotine in same brands of toothpowders (*dant manjans*) as reported in previous study. Furthermore, this year we also find it interesting to evaluate the presence of nicotine in toothpastes as well.

## 2. Materials and Methods

### 2.1. Chemicals

Eight brands of particular batches of commonly used toothpowders (*dant manjans*) and toothpastes were selected for the study from the local market. One sample from each batch was kept sealed in our laboratory as a reference sample. The toothpowders selected are coded as follows: Dabur red-M1 (8652), Vicco-M2 (008), Musaka Gul-M3 (not mentioned), Yunadent-M4 (01), Baidyanath-M5 (903), MDH-M6 (98), Gumtone-M7 (GT509), and Payokil-M8 (1503). The toothpastes selected for study were as follows: Dabur red-P1 (BD0698), Vicco-P2 (065), Arodent-P3 (64), Babool-P4 (1498), Herbodent-P5 (20), Colgate Herbal-P6 (B24CP), Ipco-P7 (not mentioned), and Dentobac-P8 (not mentioned).

Ammonia solution of extra pure quality was purchased from S.D. Fine-Chem. Ltd., Mumbai, chloroform and methanol of HPLC grade from Merck Ltd., Mumbai, and (−)-Nicotine of synthesis grade from Merck-Schuchardt, Hohebrunn, Germany. Analyses were performed with Gas chromatography Thermo Electron Corporation with MS instrument.

### 2.2. Extraction and Isolation of Nicotine from Toothpowders and Toothpastes

Extraction and isolation of nicotine from toothpowders were done as described by Agrawal and Rajagopal [6]. Extraction and isolation of nicotine from toothpastes were done by taking 25 g of toothpaste in a conical flask, 15 mL NH_3_ (Strong) was added to completely humidify the sample followed by 250 mL chloroform, and the conical flask was closed with a cap. This is kept in mechanical shaker for about one hour. After that, 2 min hand shaking and then chloroform extract is then filtered out and dried [19]. The yield values of alkaloids obtained are 0.154, 0.44, 0.22, 0.12, 0.14, 0.24, 0.56, and 0.37, respectively.

### 2.3. Method for Identification and Quantification of Nicotine by GC-MSD

All mass spectra were obtained with MS, Thermo Electron Corporation. The ion source was operated in the electron ionization mode (EI; 70 eV, 150°C). Full-scan mass spectra (*m/z* 40–450) were recorded for analyte identification. Separation was achieved with a BP fused-silica capillary column with 5% diphenyl-Poly (dimethylsiloxane (BP 5), ~30 m length, 0.25 mm i.d., 1.0 *μ*m film thickness. Samples were injected in the split mode with a split ratio of 1 : 10. The flow rate of helium as carrier gas was 1.0 mL/min. The GC operating parameters were as follows: injector temperature, 250°C; transfer line temperature, 280°C; oven temperature, programmed from 100°C (held for 3 min) at 10°C/min to 280°C (held for 3 min). The ion selected for quantification by SIM was *m/z* 162.

## 3. Results

The presence of nicotine in toothpowders (*dant manjans*) and toothpastes were confirmed by gas chromatography-mass spectroscopy (GC-MS) in full scan mode and selective ion mode (SIM).

### 3.1. Qualitative Analysis of Toothpowders and Toothpastes

**Figure 1:**
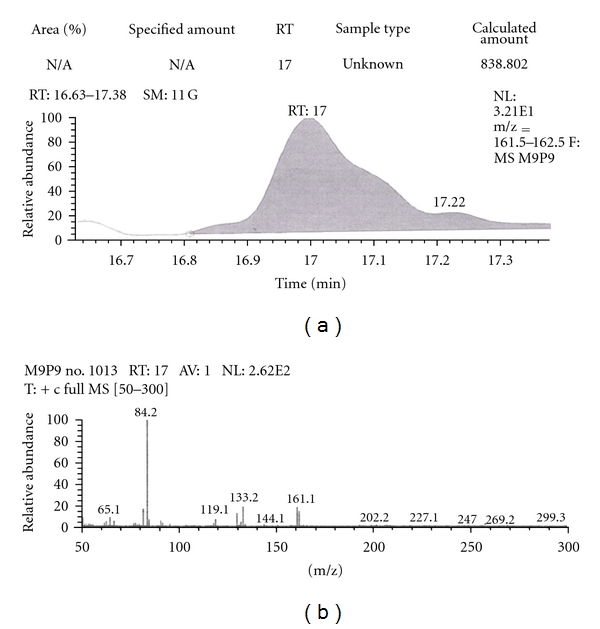
GC-MS of nicotine in full scan mode.

**Figure 2:**
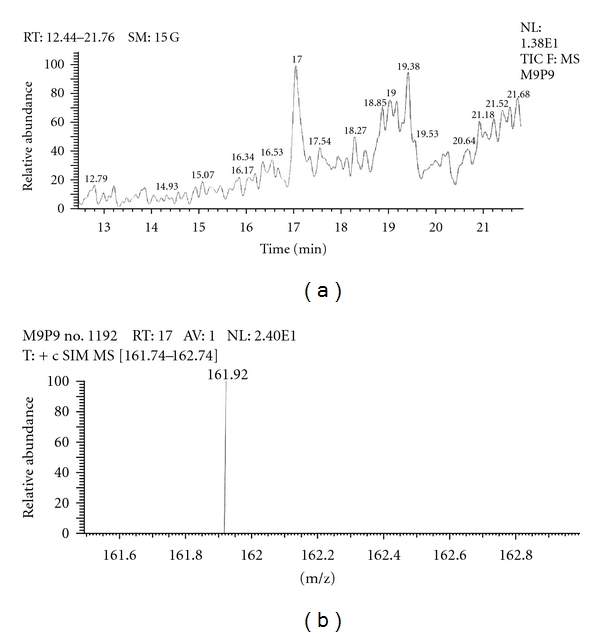
GC-MS of nicotine in SIM mode.

**Figure 3:**
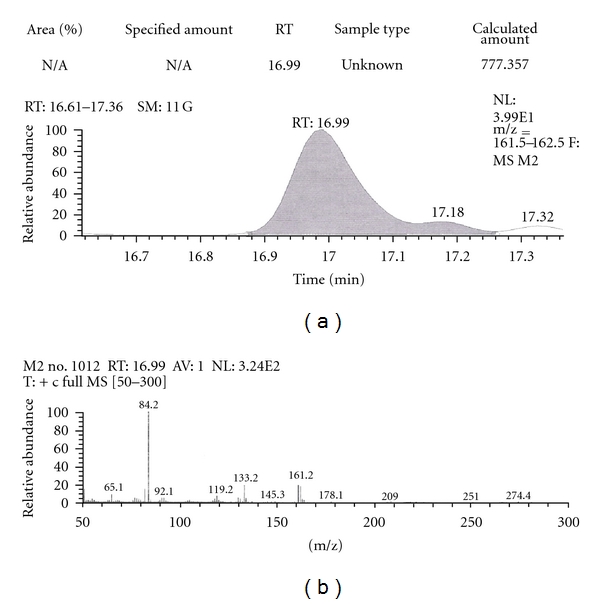
GC-MS of toothpowder M2 in full scan mode.

**Figure 4:**
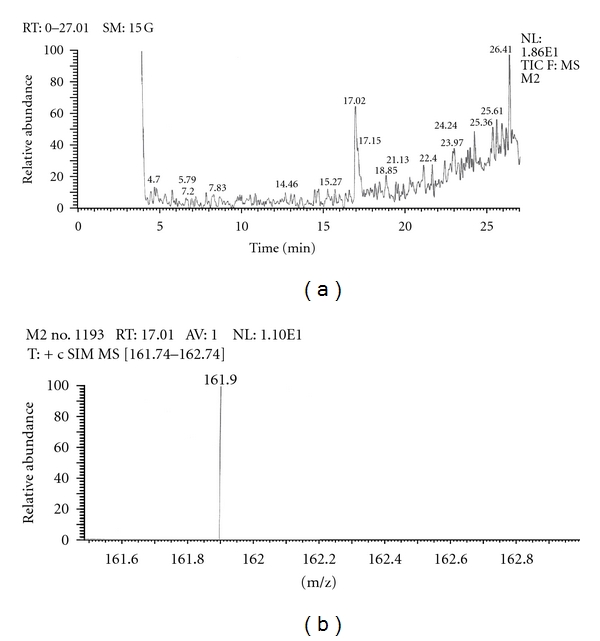
GC-MS of toothpowder M2 in SIM mode.

**Figure 5:**
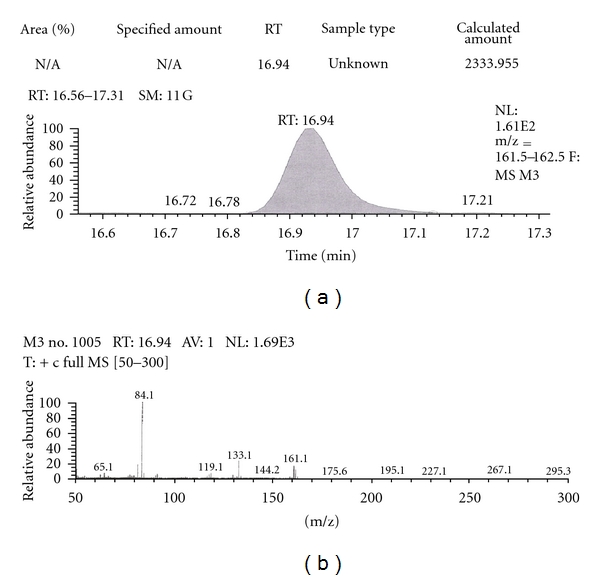
GC-MS of toothpowder M3 in full scan mode.

**Figure 6:**
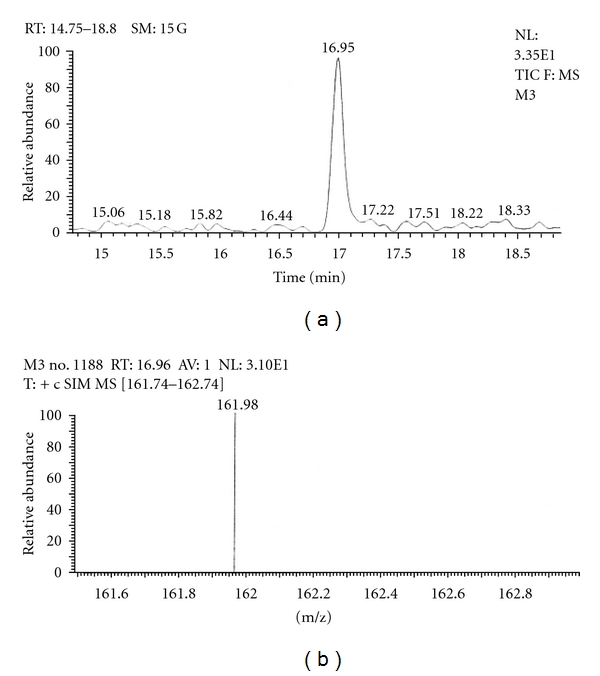
GC-MS of toothpowder M3 in SIM mode.

Out of the eight brands of toothpowders included in the present study only, two brands were found to contain nicotine. A comparison of retention time, molecular ion peak, and base peak shows the presence of nicotine in these *dant manjans* (M2 and M3). Retention time of nicotine was found to be 17.00 min. Nicotine presence was identified by comparing the RT values of toothpowder extracts with standard nicotine. The primary target ion of nicotine has a mass of 162 *m/z* [20]. The molecular ion peak at 162 *m/z* and base peak at 84 *m/z* corresponds to the fragmentation of nicotine. The RT of M2 and M3 was found to be 16.99 and 16.94, respectively. This was further confirmed by Mass spectrum (Figures 1, 2, 3, 4, 5, and 6).

**Figure 7:**
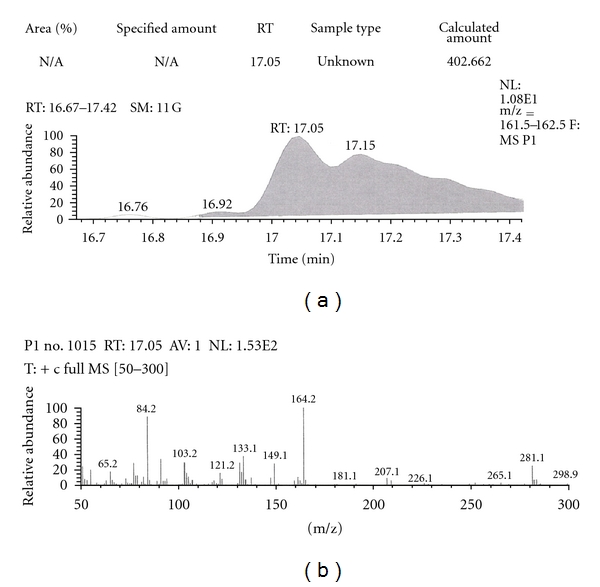
GC-MS of toothpaste P1 in full scan mode.

**Figure 8:**
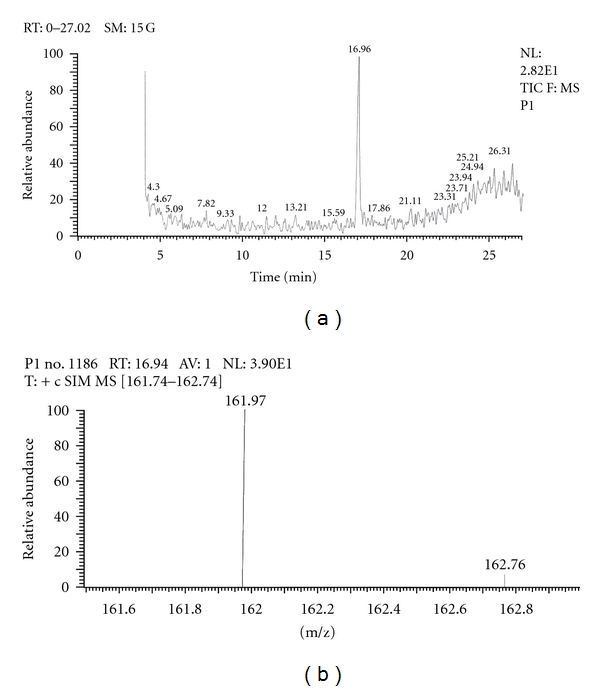
GC-MS of toothpaste P1 in SIM mode.

**Figure 9:**
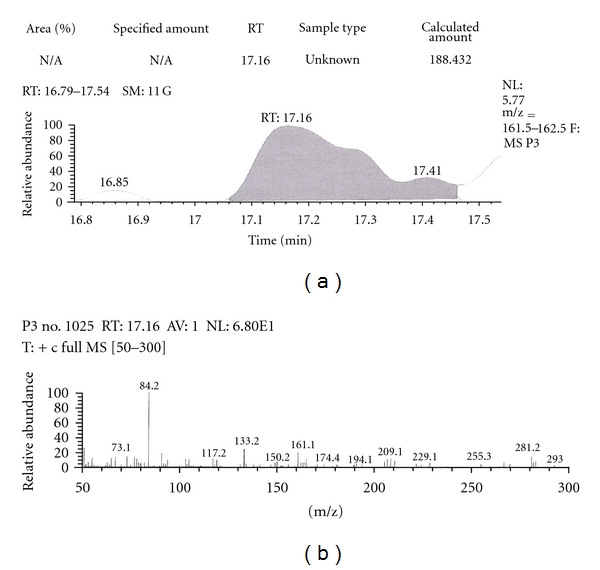
GC-MS of toothpaste P3 in full scan mode.

**Figure 10:**
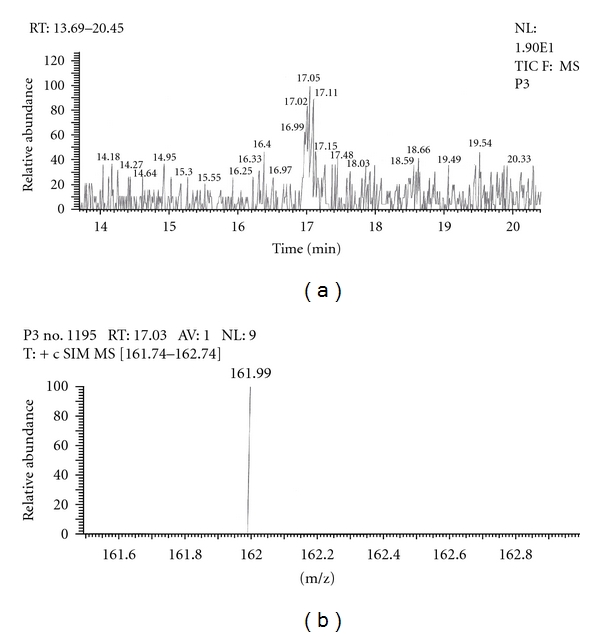
GC-MS of toothpaste P3 in SIM mode.

**Figure 11:**
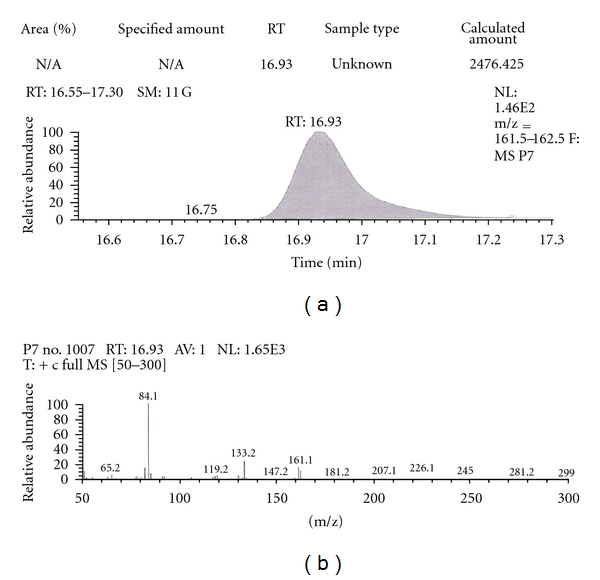
GC-MS of toothpaste P7 in full scan mode.

**Figure 12:**
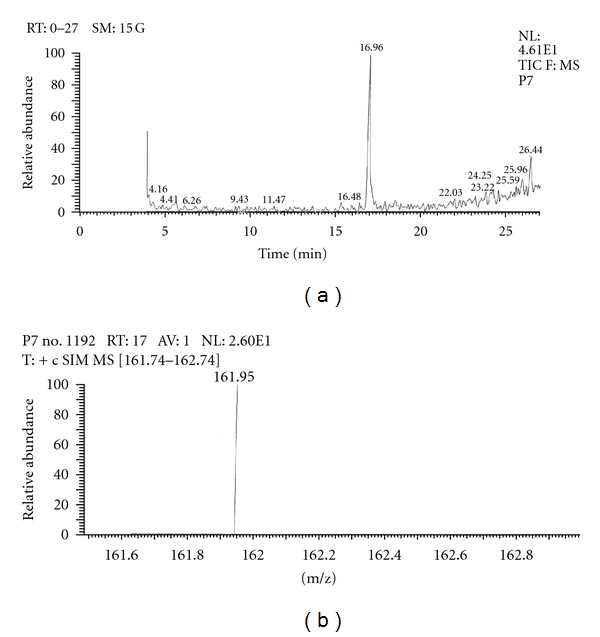
GC-MS of toothpaste P7 in SIM mode.

**Figure 13:**
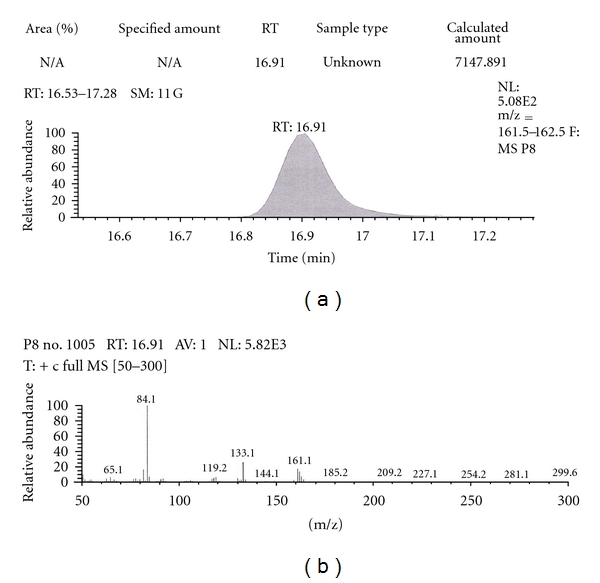
GC-MS of toothpaste P8 in full scan mode.

**Figure 14:**
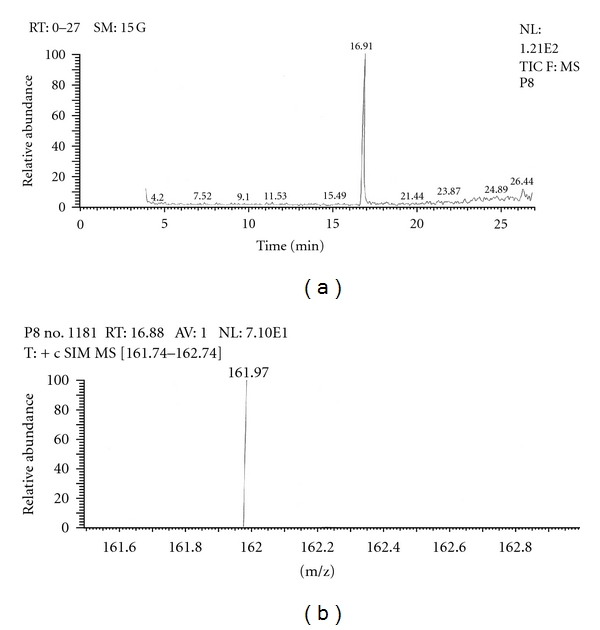
GC-MS of toothpaste P8 in SIM mode.

Similarly, Out of the eight brands of toothpastes analysed, four brands were found to contain nicotine. The RT of P1, P3, P7, and P8 was found to be 17.05, 17.16, 16.93, and 16.91, respectively as compared to that of nicotine standard 17.00 and was confirmed by its molecular ion peak and base peak (Figures 7, 8, 9, 10, 11, 12, 13, and 14).

### 3.2. Quantitative Analysis of Toothpowders and Toothpastes

The *dant manjan* extracts which indicated the presence of nicotine were also quantified with GCMS. The nicotine contents determined in GCMS of M1 and M2 were 12.95 mg/g and 216.10 mg/g respectively. Similarly, the nicotine content in toothpaste extracts of P1, P3, P7, and P8 were found to be 5.752 mg/g, 2.093 mg/g, 123.82 mg/g, and 119.13 mg/g, respectively.

## 4. Discussion

Abuse of tobacco, like drug and alcohol abuse, is a worldwide public health problem [21]. India's tobacco problem is more complex than probably that of any other country in the world, with a large consequential burden of tobacco-related disease and death [13]. Various tobacco products are popular as a dentifrice in different parts of India [16, 22]. Tobacco was not, perhaps, used for cleansing the teeth, but more so to get “kick” out of the contents of tobacco, and many companies exploit the addictive nature of nicotine by packaging and positioning them as dentifrice [22]. Researches comparing herbal toothpastes with conventional dentifrices, however, suggest that there is no specific advantage of herbal-based pastes over conventional pastes [23–25].

Indian legislation prohibits the use of tobacco as an ingredient in dental care products [6, 15]. After the passage of a law banning the use of tobacco in dental care products, the listing of tobacco as an ingredient was stopped [26]. These products, however, continue to be available openly in the market, often without mentioning tobacco as one of the ingredient [6, 16, 17].

Nicotine is a CNS stimulant, which leads to habit formation subsequently dependence on its users. Nicotine causes withdrawal symptoms such as anger, anxiety, depressed mood, difficulty in concentration, and craving for tobacco. There is strong and compelling evidence that oral tobacco use leads to oral mucosal lesions [7] including oral precancerous lesions, gingival recession [9], oral cancer [27, 28], and overall mortality. The risk increases with the duration and frequency of the habit [29].

The present studies were motivated by an interest in examining the presence of nicotine in these dentifrices and to endow with an overview of the tobacco problem in Indian dentifrices, from public health challenges to policy responses. With this background, an attempt was made to further curb the menace of tobacco in commercially available dentifrices in India and to provide a credible basis for evolving future tobacco regulation in dentifrices. Our team again analysed the same brands as of yesteryears with an objective to review that whether the regulations with respect to tobacco/nicotine in toothpowders have been adequately implemented or not. Also it was thought to estimate nicotine in toothpastes of herbal/ayurvedic origin.

In consistent with the previous report, the presence of nicotine in four out of eight brands of *dant manjans* analysed [6], this year only two bands were found to contain nicotine and is quantified as M2 (12.95 mg/g) and M3 (216.10 mg/g), respectively. This year out of the eight most commonly available herbal toothpastes selected for the study, four brands were found to contain nicotine and was quantified as P1 (5.32 mg/g), P3 (2.093 mg/g), P7 (119.13 mg/g), and P8 (123.82 mg/g), respectively. Moreover, these nicotine-containing toothpowders (*dant manjans*) and toothpastes lack pictorial warning which implies that these manufactures are disguising it as a dentifrice.

A person on a regular oral hygiene uses 2-3 g of toothpowder/paste each day [30]. The LD50 of nicotine in mice, rats, and human is 3 mg, 50 mg, and 40–60 mg, respectively. Thus, the present study indicates that a considerable amount of nicotine (2–216 mg) could enter the oral cavity if these toothpowders and toothpastes were used. Adults remove most of the toothpowder/paste from the oral cavity by rinsing. Children may retain a much greater percentage of dentifrice than adults, because of incomplete rinsing thus resulting in ingestion [30]. Once a person is addicted to nicotine, quitting is very difficult no matter whichever form he uses it. In a survey, women were found to be addictive to tobacco paste than males [31]. Thus the presence of nicotine in these dentifrices even in low concentrations could considerably lead to addiction and oral mucosal lesions [7] due to its routine use. Hence this epidemic of addiction to nicotine among young people may have enormous implications in public health [32].

Therefore, this study underscores the importance of support of prevention of tobacco use in developing countries especially India where population is roaring; tobacco use threatens to contribute to a growing proportion of the burden of disease. Thus, even in low concentrations, one would expect tobacco to be present in these dentifrices to exert its addictive influence, which emphasizes the need for strong economic, legislative, and educational means to eradicate this menace. However, much more needs to be done including possibly enacting legislation to stop the manufacturing of such dentifrices. The findings of this study will help to design, implement, and evaluate tobacco control and prevention programmes. Thus, our study clearly confirmed the presence of nicotine in few dentifrices, and regulations have not been implemented properly even after 17 years. Such issues should be effectively addressed by the regulatory authorities in India and worldwide.

## 5. Conclusion

With a population of over 1 billion people, tobacco use contributes to India's and global public health threat. Our new findings substantiate the presence of nicotine in a variety of dentifrices. Thus even after 17 years of amendment of Drugs and Cosmetic Act banning tobacco toothpowders and toothpastes, nicotine is still present in some dentifrices which clearly shows that the law is not strictly enforced and implemented. Hence regulatory authorities in India and internationally should take adequate measures to stop the addition of nicotine or tobacco to the dentifrices to provide nicotine-/Tobacco-free dentifrices. However, continued surveillance of such dental products is necessary in order to better inform and educate the public regarding the risks associated with respect to this public health concern.
